# Pilot study comparing the Childhood Arthritis & Rheumatology Research Alliance (CARRA) systemic Juvenile Idiopathic Arthritis Consensus Treatment Plans

**DOI:** 10.1186/s12969-017-0157-1

**Published:** 2017-04-11

**Authors:** Yukiko Kimura, Sriharsha Grevich, Timothy Beukelman, Esi Morgan, Peter A. Nigrovic, Kelly Mieszkalski, T Brent Graham, Maria Ibarra, Norman Ilowite, Marisa Klein-Gitelman, Karen Onel, Sampath Prahalad, Marilynn Punaro, Sarah Ringold, Dana Toib, Heather Van Mater, Jennifer E. Weiss, Pamela F. Weiss, Laura E. Schanberg, for the CARRA Registry Investigators, L. Abramson, E. Anderson, M. Andrew, N. Battle, M. Becker, H. Benham, T. Beukelman, J. Birmingham, P. Blier, A. Brown, H. Brunner, A. Cabrera, D. Canter, D. Carlton, B. Caruso, L. Ceracchio, E. Chalom, J. Chang, P. Charpentier, K. Clark, J. Dean, F. Dedeoglu, B. Feldman, P. Ferguson, M. Fox, K. Francis, M. Gervasini, D. Goldsmith, G. Gorton, B. Gottlieb, T. Graham, T. Griffin, H. Grosbein, S. Guppy, H. Haftel, D. Helfrich, G. Higgins, A. Hillard, J. R. Hollister, J. Hsu, A. Hudgins, C. Hung, A. Huttenlocher, N. Ilowite, A. Imlay, L. Imundo, C. J. Inman, J. Jaqith, R. Jerath, L. Jung, P. Kahn, A. Kapedani, D. Kingsbury, K. Klein, M. Klein-Gitelman, A. Kunkel, S. Lapidus, S. Layburn, T. Lehman, C. Lindsley, M. Macgregor-Hannah, M. Malloy, C. Mawhorter, D. McCurdy, K. Mims, N. Moorthy, D. Morus, E. Muscal, M. Natter, J. Olson, K. O’Neil, K. Onel, M. Orlando, J. Palmquist, M. Phillips, L. Ponder, S. Prahalad, M. Punaro, D. Puplava, S. Quinn, A. Quintero, C. Rabinovich, A. Reed, C. Reed, S. Ringold, M. Riordan, S. Roberson, A. Robinson, J. Rossette, D. Rothman, D. Russo, N. Ruth, K. Schikler, A. Sestak, B. Shaham, Y. Sherman, M. Simmons, N. Singer, S. Spalding, H. Stapp, R. Syed, E. Thomas, K. Torok, D. Trejo, J. Tress, W. Upton, R. Vehe, E. von Scheven, L. Walters, J. E. Weiss, P. F. Weiss, N. Welnick, A. White, J. Woo, J. Wootton, A. Yalcindag, C. Zapp, L. Zemel, A. Zhu

**Affiliations:** 1 Pediatric rheumatology, Joseph M. Sanzari Children’s Hospital, Hackensack University Medical Center, 30 Prospect Ave, Hackensack, NJ 07601 USA; 2 Pediatric rheumatology, Seattle Children’s Hospital, Seattle, USA; 3 Pediatric rheumatology, University of Alabama at Birmingham, Birmingham, USA; 4 Rheumatology, Cincinnati Children’s Hospital Medical Center, Cincinnati, USA; 5 Rheumatology, Immunology and Allergy, Brigham and Women’s Hospital, Boston, USA; 6 Childhood Arthritis & Rheumatology Research Alliance, Milwaukee, USA; 7 Pediatric rheumatology, Vanderbilt University, Nashville, USA; 8 Pediatric rheumatology, Children’s Mercy Hospital, Kansas City, USA; 9 Pediatric rheumatology, Children’s Hospital at Montefiore, Bronx, NY USA; 10 Pediatric rheumatology, Ann & Robert H. Lurie Children’s Hospital of Chicago, Chicago, USA; 11 Pediatric rheumatology, University of Chicago, Chicago, USA; 12 Pediatric rheumatology, Emory University School of Medicine, Atlanta, USA; 13 Pediatric rheumatology, Texas Scottish Rite Hospital, Dallas, USA; 14 Pediatric rheumatology, St. Christopher’s Hospital, Philadelphia, USA; 15 Pediatric rheumatology, Duke University, Durham, NC USA; 16 Pediatric rheumatology, Children’s Hospital of Philadelphia, Philadelphia, USA

**Keywords:** Systemic Juvenile Idiopathic Arthritis, Still’s disease, Biologic response modifiers, Comparative effectiveness, Pediatric rheumatology, Registries

## Abstract

**Objectives:**

To assess the feasibility of studying the comparative effectiveness of the Childhood Arthritis and Rheumatology Research Alliance (CARRA) consensus treatment plans (CTPs) for systemic Juvenile Idiopathic Arthritis (JIA) using an observational registry.

**Methods:**

Untreated systemic JIA patients enrolled in the CARRA Registry were begun on one of 4 CTPs chosen by the treating physician and patient/family (glucocorticoid [GC] alone; methotrexate [MTX] ± GC; IL1 inhibitor [IL1i] ± GC; IL6 inhibitor [IL6i] ± GC). The primary outcome of clinical inactive disease (CID) without current GC use was assessed at 9 months. Trial registration: clinicaltrials.gov NCT01697254; first registered 9/28/12 (retrospectively enrolled).

**Results:**

Thirty patients were enrolled at 13 sites; eight patients were started on a non-biologic CTP (2 GC, 6 MTX) and 22 patients on a biologic CTP (12 IL1i, 10 IL6i) at disease onset. Demographic and disease features were similar between CTP groups. CTP choice appeared to segregate by site preference. CID off GC was achieved by 37% (11 of 30) including 11/22 (50%) starting a biologic CTP compared to 0/8 starting a non-biologic CTP (*p* = 0.014). There were four serious adverse events: two infections, one appendicitis and one macrophage activation syndrome.

**Conclusions:**

The CARRA systemic JIA CTP pilot study demonstrated successful implementation of CTPs using the CARRA registry infrastructure. Having demonstrated feasibility, a larger study using CTP response to better determine the relative effectiveness of treatments for new-onset systemic JIA is now underway.

**Electronic supplementary material:**

The online version of this article (doi:10.1186/s12969-017-0157-1) contains supplementary material, which is available to authorized users.

## Background

Systemic Juvenile Idiopathic Arthritis (JIA) is a rare childhood inflammatory disease associated with significant morbidity, and characterized by arthritis accompanied by high spiking fevers, plus additional features such as rash, generalized lymphadenopathy, hepatosplenomegaly, and serositis [1]. There is considerable variation in systemic JIA treatment, due in part to a heterogeneous and somewhat unpredictable disease course, differences in physician practices, and until very recently, a lack of clinical trial data [2–5] and evidence based guidelines [6, 7]. Recent trials of IL-1 and IL-6 inhibitors (IL1i and IL6i) which demonstrated striking efficacy in systemic JIA were conducted in children with established chronic disease, so little evidence exists for use of biologic agents in new-onset or untreated systemic JIA. Nevertheless, many pediatric rheumatologists currently treat with IL1i and/or IL6i early in the course of disease [8–10]. Intriguing data from several uncontrolled case series suggest that early use of these biologic agents might favorably alter the potentially devastating outcomes of chronic persistent systemic JIA [10, 11]. On the other hand, as many as 10–40% of patients may have a monocyclic course which remits spontaneously within the first year [12, 13], complicating the evaluation of treatment effectiveness in early disease. An additional consideration is the question of whether rare, potentially fatal cardiopulmonary complications may be associated with the increased use of these agents specifically in systemic JIA [14].

With new options for treatment now commercially available and FDA approved, there is an urgent need to determine the relative effectiveness, safety and tolerability of commonly used treatments in systemic JIA. While a blinded randomized multi-arm controlled clinical trial would be ideal, it would be cost-prohibitive, require a very large sample size and likely not be feasible to perform in such a rare disease of childhood. Comparative effectiveness research (CER) studies in an observational, routine care setting are therefore a more practical approach to examine which treatments are effective and most appropriate for an individual child [15, 16]. In such a study, physician choice (together with patient/caregiver preferences) would be the primary determining factor for treatment selection. Such a study could be successful if there were equipoise between most common treatments and if the treatments and data collection were standardized and captured at predetermined intervals. The development of Consensus-derived standardized Treatment Plans (CTPs) for systemic JIA funded by a National Institute of Arthritis and Musculoskeletal and Skin Diseases (NIAMS) Challenge Grant (1RC1AR058605-01) to the Childhood Arthritis and Rheumatology Research Alliance (CARRA) was a key step in this process and in improving outcomes for patients with systemic JIA [17, 18]. The methods by which the CARRA CTPs were developed using CARRA-wide surveys to identify the current most commonly used treatments for systemic JIA and the standardization process using consensus methodology are outlined in a previously published article [17].

A pilot study comparing the published CARRA systemic JIA CTPs was funded by an Arthritis Foundation Innovative Research Grant and conducted in a limited number of sites in order to test the acceptance and usability of these CTPs in preparation for a future large comparative effectiveness study that would utilize the broader CARRA Registry network. This pilot study was conducted to assess the feasibility of conducting a larger more definitive CER study and to identify possible issues that could arise in such a study [19].

## Methods

Patients were enrolled in the CARRA Registry and treated with one of the systemic JIA CTPs at 13 CARRA Registry sites for 9 months during the period from 2011 to 2014. Funding from NIAMS via the American Recovery and Reinvestment Act (ARRA) in 2010 enabled the CARRA Registry, a multi-center prospective observational study of children with pediatric rheumatic diseases including JIA. Altogether, investigators enrolled over 9,500 patients in what is now called the CARRA Legacy Registry, including more than 7,000 with JIA (>500 with systemic JIA) at 60 sites over 3 years [9]. The CARRA Registry general protocol and consent was approved by the Duke University IRB (#Pro00054616) and at all participating site IRBs. Because the CTP study is not interventional and patients received standard-of-care therapy at the discretion of their treating physician, additional consent for participation beyond this standard consent for the CARRA Registry was not required.

The entry criteria used were a CARRA-modified version of the International League of Associations of Rheumatology (ILAR) criteria [20], as published in the original article describing the development of the systemic JIA CTPs [17]. In order to capture as many children with systemic JIA as possible while avoiding inclusion of children with self-limited febrile illnesses or alternative diagnoses, it was decided by consensus of the pediatric rheumatologists in the CARRA JIA Research Committee to adapt the ILAR criteria for new-onset patients. The modifications were to require at least 2 weeks of fever (but not necessarily on continuous days), at least one joint with physician-documented arthritis for at least a week, along with at least one other ILAR systemic JIA feature. No prior treatment for systemic JIA aside from NSAIDs or short-term (less than 2 weeks) GC were allowed. There was no randomization or blinding of treatment assignment; instead, a CTP was chosen for each child by the treating pediatric rheumatologist after discussion with the child and family as part of customary clinical decision making process. The CTP choices were: (1) GC only; (2) Methotrexate ± GC; (3) IL1i (either anakinra or canakinumab) ± GC [18]; and (4) IL6i (tocilizumab) ± GC.

Follow-up visits occurred at intervals defined by the CTPs and consistent with routine care: 2 weeks, 1, 3, 6 and 9 months after baseline. Unscheduled visits were conducted if there was a change in CTP medication. Clinical and laboratory data, determined previously by consensus-based process and also consistent with routine care, were collected at these time points through the CARRA Registry as outlined in the CTPs [17]. The CTPs recommended that if patients worsened or failed to improve sufficiently as determined by the physician’s assessment of disease activity, GC could be added or increased, or the treatment (CTP) changed. Suggested GC tapering schedules corresponding to very rapid, rapid, moderate, and slow tapering schedules were provided to be used by the treating physician but were not compulsory. By 3 months after CTP initiation, if the child was still receiving ≥50% of the starting dose of GC, it was recommended that the treatment (CTP) should be changed anticipating a better outcome. The treating physicians were asked why a particular initial CTP was chosen, and to provide the reason if the treating CTP was changed. Serious adverse events, including macrophage activation syndrome (MAS), and side effects of medications, were collected.

Outcomes included the primary endpoint of the study: clinical inactive disease (CID) off glucocorticoids at 9 months. CID was defined using the ACR provisional criteria for defining clinical inactive disease which specifies all the following: no active arthritis, a physician’s global assessment of disease activity score of 0, ESR and/or CRP in the normal range, no features of systemic JIA (fever, rash, serositis, splenomegaly, or generalized lymphadenopathy), no uveitis and duration of morning stiffness for <15 min [21]. All inactive disease criteria [22] were available for all children who had a documented 9 month visit. For the primary endpoint analysis, all patients were included even if lost to follow-up. The GC and methotrexate CTP-initiated patients were combined as the non-biologic CTP group, while the IL1i and IL6i CTP initiated patients were combined as the biologic CTP group. Secondarily, all 4 CTPs were analyzed separately assessing achievement of the primary endpoint, as well as the ability to lower GC doses.

Safety assessments included reporting of serious adverse events (SAEs), defined as an adverse event that results in death, is life-threatening, requires hospitalization or prolongation of a hospitalization, persistent or significant disability or in capacity, or a congenital anomaly or birth defect; and adverse events (AEs). AEs were graded using Common Terminology Criteria for Adverse Events (CTCAE v4.0). Grade 1 AE was asymptomatic or with mild symptoms and required either clinical or diagnostic observations or no intervention. Grade 2 AE was defined as moderate severity requiring minimal, local or noninvasive intervention. Grade 3 was a severe or medically significant event but not immediately life-threatening and either hospitalization or prolongation of hospitalization were required. Grade 4 AEs were defined as those with life-threatening consequences or requiring urgent intervention. The MedDRA dictionary was not used for safety reporting in this pilot study.

Differences in data collected from the CTPs were compared using chi square, Fisher’s exact, and Wilcoxon rank sum tests.

## Results

Thirty untreated, mostly newly diagnosed systemic JIA patients were enrolled into the CARRA Registry at 13 sites and started on a CARRA systemic JIA CTP. The children’s clinical and demographic characteristics are summarized in Table 1.

**Table 1:** Baseline demographic and disease characteristics in all systemic JIA CTP patients

	GC (*N* = 2)	MTX (*N* = 6)	IL1 Inhibitor (*N* = 12)	IL6 Inhibitor (*N* = 10)	Total (*N* = 30)	*P*-value
Age in years^a^	12.8	3.4	4.0	9.8	5.7	0.047
Female	50%	67%	92%	90%	83%	0.3
White	0%	83%	33%	80%	57%	0.03
Disease duration (days)^a^	121	38	31	55	42.5	0.2
Polyarthritis	0%	50%	67%	80%	63%	0.18
Number of active joints^a^	1.0	4.5	5.0	5.5	4.0	0.28
ESR >3x ULN	100%	67%	91%	43%	73%	0.27
CRP >3x ULN	100%	83%	91%	40%	79%	0.28
Ferritin (ng/mL)^a^	12,874	1,394	1,062	627	783	0.09
CHAQ score^a^	0.9	1.3	1.9	0.8	1.4	0.6
Pain score (0–10)^a^	3	6.5	6	7.5	6	0.6
Physician global assessment (0–10)^a^	4	7	5	5	5.5	0.12
Parent global assessment well-being (0–10)^a^	0	7	6	6	6	0.103
Initiated GC at baseline	100%	100%	42%	70%	67%	0.029
Initial GC dose (mg/kg)^a^	0.67	0.97	0.00	0.68	0.80	0.548
GC prior to baseline	50%	16.7%	8.3%	30%	20%	0.350
Initial dose of each CTP medication in mg/kg (IQR)^a^	0.67 (0.6–0.73)	0.487 (0.46–0.64)	2.93 (2–3.6)^b^	8.14 (8–12)	Not Applicable	

^a^Median

^b^Anakinra dose (anakinra was used as the initial IL-1i in all patients)

Five patients were taking GC prior to starting their respective CTP (median 12 days prior to baseline): of these patients, three patients started on IL6i, and one each started on IL1i and MTX. One patient started on the IL1i CTP was lost to follow-up after the baseline visit.

**Fig. 1:**
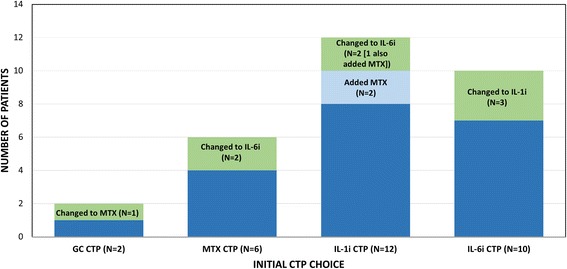
Initial Consensus Treatment Plan (CTP) Choices and Subsequent Treatment Changes. The total numbers of patients started on each CTP are shown, along with those remaining on the original CTP medication (*dark blue*), switching to a different CTP (*green*) or adding another CTP medication (*light blue*). IL1i: IL1 inhibitor; IL6i: IL6 inhibitor

**Fig. 2:**
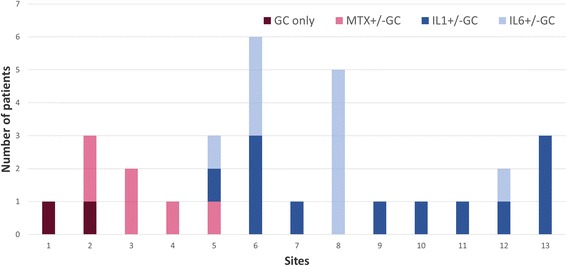
Initial Consensus Treatment Plan (CTP) Choice By Site. CTP choices distributed by site, showing that 4 sites started only a non-biologic CTP (*red colors*), while 8 sites started only a biologic CTP (*blue colors*), IL1i: IL1 inhibitor; IL6i: IL6 inhibitor

Figure 1 shows the number of children in each CTP group that eventually switched CTPs or added a DMARD such as methotrexate. Ultimately, 12 of 30 children (40%) switched from their original CTP or added methotrexate. Figure 2 shows initial CTP choice by site. Sites 1–4 exclusively used non-biologic CTPs to start, while sites 6–13 exclusively used biologic CTPs, with site 5 the only site to start both biologic and non-biologic CTPs. Although the majority of sites enrolled few patients (1 to 3), reasons for selecting a particular CTP varied mostly by site rather than CTP. The most common reasons cited were: “treatment works best” in 19/29 (65.5%) (GC, IL1i, IL6i CTPs); “treatment is safer” in 15/29 (51.7%) (MTX, IL1i, IL6i); “treatment is better tolerated” in 14/29 (48.3%) (MTX, IL1i, IL6i); and “always select(s) this treatment” in 10/29 (34.5%) (GC, IL1i, IL6i).

Owing to the reliance on data collection at regular office visits, there was variability in the designated 9 month visit dates with the median being 39.9 weeks after baseline (IQR: 37.3, 43.4, range 29.9–54.7 weeks) Three children did not have a 9 month visit (1 GC and 2 IL1i CTP patients); of these, 1 of the 2 IL1i treated patients and the GC treated patient were lost to follow-up.

While feasibility of the study methodology rather than clinical outcomes was the major purpose of this study, and with the caveat that no attempt was made to adjust for patient characteristics between these very small groups, differences between non-biologic and biologic CTPs were nevertheless observed. The primary endpoint of CID off GC at 9 months was achieved by 50% of biologic CTP initiated children (11 of the 22 children), whereas none of the eight non-biologic CTP initiated children achieved this endpoint (*p* = 0.014) (Table 2).

**Table 2:** Achievement of Clinical Inactive Disease (CID) off Glucocorticoids (GC) in Non-Biologic and Biologic initiated patients (primary endpoint), as well as in the 4 Consensus Treatment Plan (CTP) groups

							Biologic grouping		
Patient Status at Month 9	Total *N* = 30	Gluco-corticoids *N* = 2	Methotrex-ate *N* = 6	IL1i^a^ *N* = 12	IL6i^a^ *N* = 10	*p*-value	Non-Biologic CTP *N* = 8	Biologic CTP *N* = 22	*p*-value
CID	13/30 (43.3%)	0/2	2/6 (33.3%)	5/12 (41.7%)	6/10 (60.0%)	0.549	2/8 (25.0%)	11/22 (50.0%)	0.407
Off GC	20/30 (66.7%)	1/2 (50.0%)	1/6 (16.7%)	10/12 (83.3%)	8/10 (80.0%)	0.019	2/8 (25.0%)	18/22 (81.8%)	0.007
CID off GC	11/30 (36.7%)	0/2	0/6	5/12 (41.7%)	6/10 (60.0%)	0.079	0/8	11/22 (50.0%)	0.014
CID off GC and no CTP change	8/30 (26.7%)	0/2	0/6	3/12 (25.0%)	5/10 (50.0%)	0.168	0/8	8/22 (36.4%)	0.071

Also shown are all patients who achieved CID (regardless of GC), all patients who were off GC, and all patients who achieved CID off GC and did not change their CTP treatments, at 9 months. Patients lost to follow-up were considered treatment failures

^a^IL1i: IL-1 inhibitor; IL6i: IL-6 inhibitor

This failure to achieve CID off GC was not simply due to slow tapering of GC: 6 of 8 (75%) children started on non-biologic CTPs had active disease at 9 months compared with 9 of 22 (41%) of children started on biologic CTPs (*p* = 0.407); and 6 of 8 (75%) of non-biologic CTP treated children were still taking GC at 9 months, compared to 4 of 22 (18%) of biologic CTP treated children (*p* = 0.007). Table 3 also shows the primary endpoint analysis by individual CTP group, as well as of patients who achieved the endpoint without switching/adding CTP medications. Likely due to the small numbers in each group, statistical significance was not achieved in these groups. All but one provider generally followed the CTP GC tapering recommendations, so that if by Month 3, if GC were not tapered to less than 50% of the initial dose, treatment was intensified or the CTP was changed. There was no significant difference in the median number of days from baseline of the 9 month visit between the biologic (282 days) and non-biologic (271 days) CTP groups (*p* = 0.599), despite the variability in the 9 month visit date discussed above.

**Table 3:** Adverse events reported in all patients by CTP at time of event

CTP at time of AE	GC	MTX	IL-1 Inhibitor	IL-6 Inhibitor	Total
Grade 2	0	2 (allergic reaction [1], arthritis flare [1])	5 (MAS [1], hepatitis [1], rash [1], Strep pharyngitis [1], injection site reaction [1])	8 (fever [1}, rash [1], arthritis flare [2], headache [1], neutropenia [1], viral illness [1], infusion reaction [1])	15
Grade 3	0	0	2 (infections)^b^	0	2
Grade 4	0	0	0	1 (infusion reaction)	1
SAE	0	1^a^	2 (infections)^b^	1 (MAS)	3

^a^Appendicitis and appendectomy

^b^1 varicella (anakinra), 1 cellullitis (canakinumab)

Adverse events are listed in Table 3. There was one Grade 4 AE infusion reaction with tocilizumab. There were 4 SAE’s: 2 that resulted in hospitalization for intravenous antimicrobial therapy (cellulitis in a child taking canakinumab and GC [Grade 3] and varicella in a child taking anakinra [Grade 3]), 1 hospitalization for appendicitis and appendectomy on methotrexate and GC, and 1 for macrophage activation syndrome (MAS) in a child on tocilizumab (Grade 3). One additional child who was receiving anakinra developed MAS and was treated with oral prednisone without hospitalization (Grade 2). All patients recovered uneventfully.

## Discussion

This pilot study assessed the feasibility of using standardized CARRA CTPs for untreated systemic JIA, 90% of whom had disease onset less than 4 months prior to treatment, to evaluate the comparative effectiveness of commonly used treatment approaches. Importantly, the study established that the CARRA Registry and the coordinating center at the Duke Clinical Research Institute (DCRI) could effectively operationalize and capture the data for such a study, but it also demonstrated that better methods to decrease missing visits and data, and to be able to capture clinical data between visits for the larger study were needed. Interestingly, each plan was used with similar frequency aside from the GC-only plan, confirming community equipoise on treatment selection. The choice of plan varied by site/individual physician rather than disease characteristics of the individual child, with the caveat that at most a few patients were treated at each site. However, plan choice driven by site allows for a “pseudo-block randomization” in the context of a larger observational comparative effectiveness study. The distribution of CTP usage followed results of surveys conducted as part of the development of the original CTPs [17], indicating that there continues to be substantial variability in physician practice with regard to initial treatment choices for systemic JIA.

The results of this pilot study underline the need for a larger definitive comparative effectiveness study assessing all 4 CTPs. The quandary about the safest and most effective treatment of new-onset systemic JIA has been informed by studies of the IL1i anakinra (recombinant IL-1 receptor antagonist). Recent studies looking at early use of anakinra in systemic JIA demonstrated a marked reduction in the development of chronic persistent arthritis from a historical rate of 50–60% to less than 10% [9, 11], a rate of response markedly distinct from that observed in a randomized controlled trial of anakinra in established chronic systemic JIA [2]. These studies raise the possibility of a “window of opportunity” in early systemic JIA during which cytokine blockade could be especially effective [23]. Even though this pilot study included small numbers of children who were followed for only 9 months, the results indicate that untreated systemic JIA patients started on IL-1i or IL-6i biologics achieved the primary endpoint (CID off GC at 9 months) more frequently than children not started on a biologic. Children treated with non-biologic CTPs were more likely to both have continued active disease and continue to be treated with GC compared to those treated with biologic CTPs. Whether this will hold true in a larger population of children followed for a longer period of time remains to be seen, as no attempt was made to correct for baseline clinical features or other potentially confounding variables, rendering the conclusions of this this pilot study far from definitive [19].

Study limitations include the observational design. As noted, no adjustments were made to address confounding by indication (prescriber channeling). The basic CTP approach assumes that treatment choice is determined by physician preferences rather than patient disease characteristics, and further assumes that individual physicians influence disease outcomes only through the CTP choice. We observed evidence in favor of the first assumption in this pilot study, but the latter assumption is difficult to assess given the strong correlations between physician and treatment choice. Missing data points and visits outside of the suggested visit schedule were relatively common, limiting analysis of the results. Children were sometimes started on medications, especially GC, prior to starting the CTP. Short-term use of GC was allowed, which may have changed the baseline disease characteristics of these patients. However, prior treatment with GC is particularly difficult to avoid at presentation of new onset systemic JIA, a phase of the disease in which children are often acutely ill; excluding these children could eliminate significant numbers of children, particularly the sickest children. In addition, patients were followed for a relatively short period of time (9 months), with CID assessed at a single time point that may incompletely reflect the treatment response in this dynamic disease. Evaluation of stably inactive disease, such as clinical inactive disease for 6 consecutive months (i.e., clinical remission) and clinical inactive disease for 12 consecutive months off of all medications (i.e., clinical remission off medication) [21] will be assessed in the larger CTP study informed by this pilot, but was not able to be captured in this study. Data collection through the CARRA Registry will allow assessment of long-term outcomes for decade or more after Registry enrollment, including outcomes such as medication safety and functional status as well as remission. Lastly, the small sample size, differences in the demographics of the study cohort compared to other systemic JIA cohorts, the short follow-up duration and missing data limit the external validity of the results of this pilot study.

The use of modified ILAR criteria for diagnosis may affect the generalizability of the study results. It is recognized that many children with systemic JIA in the earliest stages of disease require treatment, yet may not strictly fulfill ILAR criteria (especially the requirement for 6 weeks of arthritis) [24]. Indeed, the Yamaguchi criteria for Adult Onset Still Disease (the adult correlate of systemic JIA) does not require overt arthritis for diagnosis [25, 26]. It is possible that use of these modified criteria by clinicians other than experienced pediatric rheumatologists may lead to misdiagnosis. We emphasize that it is critical to exclude other diagnoses that may be mistaken for systemic JIA, such as infection, malignancy (in particular acute lymphoblastic leukemia, lymphoma and neuroblastoma) or genetic auto-inflammatory diseases prior to starting treatment for systemic JIA using the CTPs, especially before treatment with GC. In cases where the diagnosis is not based on the triad of characteristic fever, rash and arthritis, the advice of an expert pediatric rheumatologist should be sought.

The larger comparative effectiveness study, implementing the systemic JIA CTP study at 60 or more CARRA Registry sites, will retain the observational study design including non-randomization of treatment allocation. This study, termed the FiRst-line Options in Systemic JIA Treatment (FROST), will address limitations recognized in the pilot study including reducing important potential biases such as confounding by indication by using propensity scoring, as well as instrumental variable and marginal structural model analyses. Bayesian analytic methods will also be utilized for the larger study. Bayesian prior belief exercises needed for the analyses were conducted at a recent CARRA annual meeting. This pilot study was employed to inform the power calculations for FROST, in which we plan to use an intention-to-treat analytic approach (see Additional files 1 and 2). Aggressive efforts to improve data quality are planned, including more rigorous site education and awareness, leveraging various forms of communication. More detailed information about GC use prior to and during treatment will be obtained and analyzed. Children/parents will be queried about symptoms and GC dose on a regular basis using home electronic mobile device reporting. We will collect detailed information about SAEs and important medical events (IME) throughout the CTP study (including MedDRA coding of events). Lastly, important long-term follow up of children started on the CARRA systemic JIA CTPs will be accomplished using existing CARRA Registry infrastructure to follow all enrolled patients for 10 or more years, including use of a call center to contact patients and their families if the child transitions from pediatric to adult care or moves to a non-participating center.

We observed a few unexpected patient characteristics in this small pilot study, such as a female predominance, whereas most large studies of systemic JIA have reported a nearly equal female to male ratio [8, 9]. There were also patients with longer than expected disease duration prior to study enrollment. These differences are anticipated to be minimized by the inclusion of many more patients into the larger FROST CTP study.

## Conclusions

This pilot study of the four CARRA standardized CTPs for untreated mostly new-onset systemic JIA successfully demonstrated the feasibility of their implementation in a larger observational comparative effectiveness study using the CARRA Registry for data collection. FROST, the planned follow-on larger, longitudinal study will establish whether biologic medications started early in the disease course will result in better outcomes for children with new onset systemic JIA.

## Additional files

Additional file 1: Power Calculations for larger comparative effectiveness research study of systemic JIA Consensus Treatment Plans [27]. (DOC 24 kb)

Additional file 2: Table S1. Scenarios evaluated for degree of imbalance in propensity score modeling. (DOC 28 kb)

## References

[1] Prakken B, Albani S, Martini A (2011). Juvenile idiopathic arthritis. Lancet.

[2] Quartier P, Allantz F, Cimaz R, Pillet P, Msesiaen C (2011). A multicentre, randomised, double-blind, placebo-controlled trial with the interleukin-1 receptor antagonist anakinra in patients with systemic-onset juvenile idiopathic arthritis (ANAJIS trial). Ann Rheum Dis.

[3] Ruperto N, Brunner HI, Quartier P, Constantin T, Wulffraat N, Horneff G, Brik R, McCann L, Kasapcopur O, Rutkowska-Sak L (2012). Two randomized trials of canakinumab in systemic juvenile idiopathic arthritis. N Engl J Med.

[4] De Benedetti F, Brunner HI, Ruperto N, Kenwright A, Wright S, Calvo I, Cuttica R, Ravelli A, Schneider R, Woo P (2012). Randomized trial of tocilizumab in systemic juvenile idiopathic arthritis. N Engl J Med.

[5] Yokota S, Imagawa T, Mori M, Miyamae T, Aihara Y, Takei S, Iwata N, Umebayashi H, Murata T, Miyoshi M (2008). Efficacy and safety of tocilizumab in patients with systemic-onset juvenile idiopathic arthritis: a randomised, double-blind, placebo-controlled, withdrawal phase III trial. Lancet.

[6] Ringold S, Weiss PF, Beukelman T, DeWitt EM, Ilowite NT, Kimura Y, Laxer RM, Lovell DJ, Nigrovic PA, Robinson AB (2013). 2013 update of the 2011 American College of Rheumatology recommendations for the treatment of juvenile idiopathic arthritis: recommendations for the medical therapy of children with systemic juvenile idiopathic arthritis and tuberculosis screening among children receiving biologic medications. Arthritis Rheum.

[7] Beukelman T, Patkar NM, Saag KG, Tolleson-Rinehart S, Cron RQ, DeWitt EM, Ilowite NT, Kimura Y, Laxer RM, Lovell DJ (2011). 2011 American College of Rheumatology recommendations for the treatment of juvenile idiopathic arthritis: initiation and safety monitoring of therapeutic agents for the treatment of arthritis and systemic features. Arthritis Care Res (Hoboken).

[8] Klotsche J, Raab A, Niewerth M, Sengler C, Ganser G, Kallinich T, Niehues T, Hufnagel M, Thon A, Hospach T (2016). Outcome and trends in treatment of systemic juvenile idiopathic arthritis in the German National Pediatric Rheumatological Database from 2000 to 2013. Arthritis Rheum.

[9] Janow G, Schanberg LE, Setoguchi S, Hasselblad V, Mellins ED, Schneider R, Kimura Y, Investigators CLR (2016). The Systemic Juvenile Idiopathic Arthritis Cohort of the Childhood Arthritis and Rheumatology Research Alliance Registry: 2010–2013. J Rheumatol.

[10] Nigrovic PA, Mannion M, Prince FH, Zeft A, Rabinovich CE (2011). Anakinra as first-line disease-modifying therapy in systemic juvenile idiopathic arthritis: report of forty-six patients from an international multicenter series. Arthritis Rheum.

[11] Vastert SJ, de Jager W, Noordman BJ, Holzinger D, Kuis W, Prakken BJ, Wulffraat NM (2014). Effectiveness of first-line treatment with recombinant interleukin-1 receptor antagonist in steroid-naive patients with new-onset systemic juvenile idiopathic arthritis: results of a prospective cohort study. Arthritis Rheum.

[12] Singh-Grewal D, Schenider R, Bayer N, Feldman BM (2006). Predictors of disease course and remission in systemic Juvenile Idiopathic Arthritis. Arthritis Rheum.

[13] Spiegel LR, Schneider R, Lang BA, Silverman ED, Laxer RM, Stephens D (2000). Early predictors of poor functional outcome in systemic-onset juvenile rheumatoid arthritis: a multicenter cohort study. Arthritis Rheum.

[14] Kimura Y, Weiss JE, Haroldson KL, Lee T, Punaro M, Oliveira S, Rabinovich E, Riebschleger M, Anton J, Blier PR (2013). Pulmonary hypertension and other potentially fatal pulmonary complications in systemic juvenile idiopathic arthritis. Arthritis Care Res (Hoboken).

[15] Sox HC, Greenfield S (2009). Comparative effectiveness research: a report from the Institute of Medicine. Ann Intern Med.

[16] Yokota S, Itoh Y, Morio T, Origasa H, Sumitomo N, Tomobe M, Tanaka K, Minota S (2016). Tocilizumab in systemic juvenile idiopathic arthritis in a real-world clinical setting: results from 1 year of postmarketing surveillance follow-up of 417 patients in Japan. Ann Rheum Dis.

[17] DeWitt EM, Kimura Y, Beukelman T, Nigrovic PA, Onel K, Prahalad S, Schneider R, Stoll ML, Angeles-Han S, Milojevic D (2012). Consensus treatment plans for new-onset systemic juvenile idiopathic arthritis. Arthritis Care Res (Hoboken).

[18] Kimura Y, DeWitt EM, Beukelman T, Stoll ML, Nigrovic PA, Onel K, Prahalad S, Angeles-Han S, Schneider R, Juvenile Idiopathic Arthritis Disease-Specific Research Committee of the Childhood A (2014). Adding canakinumab to the Childhood Arthritis and Rheumatology Research Alliance consensus treatment plans for systemic juvenile idiopathic arthritis: comment on the article by DeWitt et al. Arthritis Care Res (Hoboken).

[19] Thabane L, Ma J, Chu R, Cheng J, Ismaila A, Rios LP, Robson R, Thabane M, Giangregorio L, Goldsmith CH (2010). A tutorial on pilot studies: the what, why and how. BMC Med Res Methodol.

[20] Petty RE, Southwood TR, Manners P, Baum J, Glass DN, Goldenberg J, He X, Maldonado-Cocco J, Orozco-Alcala J, Prieur AM (2004). International League of Associations for Rheumatology classification of juvenile idiopathic arthritis: second revision, Edmonton, 2001. J Rheumatol.

[21] Wallace CA, Giannini EH, Huang B, Itert L, Ruperto N, Childhood Arthritis Rheumatology Research A, Pediatric Rheumatology Collaborative Study G, Paediatric Rheumatology International Trials O (2011). American College of Rheumatology provisional criteria for defining clinical inactive disease in select categories of juvenile idiopathic arthritis. Arthritis Care Res (Hoboken).

[22] Wallace CA, Ruperto N, Giannini E, Childhood Arthritis and Rheumatology Research Alliance; Pediatric Rheumatology International Trials Organization: Pediatric Rheumatology Collaborative Study Group (2004). Preliminary criteria for clinical remission for select categories of juvenile idiopathic arthritis. J Rheumatol.

[23] Nigrovic PA (2014). Review: is there a window of opportunity for treatment of systemic juvenile idiopathic arthritis?. Arthritis Rheum.

[24] Behrens EM, Beukelman T, Gallo L, Spangler J, Rosenkranz M, Arkachaisri T, Ayala R, Groh B, Finkel TH, Cron RQ (2008). Evaluation of the presentation of systemic onset juvenile rheumatoid arthritis: data from the Pennsylvania Systemic Onset Juvenile Arthritis Registry (PASOJAR). J Rheumatol.

[25] Yamaguchi M, Ohta A, Tsunematsu T, Kasukawa R, Mizushima Y, Kashiwagi H, Kashiwazaki S, Tanimoto K, Matsumoto Y, Ota T (1992). Preliminary criteria for classification of adult Still’s disease. J Rheumatol.

[26] Jiang L, Wang Z, Dai X, Jin X (2011). Evaluation of clinical measures and different criteria for diagnosis of adult-onset Still’s disease in a Chinese population. J Rheumatol.

[27] Lee J, Kim J, Jung SH (2007). Bayesian analysis of paired survival data using a bivariate exponential distribution. Lifetime Data Anal.

