# Supplementary material for: Pilot study comparing the Childhood Arthritis & Rheumatology Research Alliance (CARRA) systemic Juvenile Idiopathic Arthritis Consensus Treatment Plans
Source: Pediatr Rheumatol Online J. 2017 Apr 11;15:23. doi: 10.1186/s12969-017-0157-1 (PMC5387287; doi:10.1186/s12969-017-0157-1)
Supplement: Supplementary file 1 — Power Calculations for larger comparative effectiveness research study of systemic JIA Consensus Treatment Plans [27]. (DOC 24 kb) [file 12969_2017_157_MOESM1_ESM.doc]

**Additional file 1**

**Power Calculations for larger CER study of sJIA CTPs**

Using the results of the sJIA CTP pilot study, we assume usage of each strategy will be: Non-biologic (GC only and MTX CTPs): 33%; Biologic (IL-1 and IL-6 CTPs): 67%. Our sample of 200 will reflect this treatment ratio and include exactly 67 patients on non-biologic and 133 on biologic CTPs. In a stratified propensity score analysis, the required sample size depends on the degree of imbalance between groups on the calculated PS (which will not be known until patients are enrolled), the percentages in each PS stratum (which we can fix at 20% in each of 5 strata) as well as the actual CID probabilities for the two CTP groups in each propensity score stratum. The assumed overall probabilities of achieving CID off GC are 0.3 for non-biologic CTPs and 0.6 for biologic CTPs.  We carried out a simulation study to calculate the Bayesian power for the propensity-stratified comparison of the proportion with clinically inactive disease off GC in the biologic strategy vs. this same proportion in the non-biologic strategy. Here, we define a “statistically significant” benefit for the biologic CTPs as a posterior probability of at least 95% that that clinically inactive disease is more frequent with biologic CTPs.  With moderate imbalance in the PS (the ratio of non-biologics to biologics ranging from 20%:80% to 46%:54% across PS strata), there is a 99% probability of showing that biologic CTPs are significantly more effective than non-biologic CTP (i.e., the difference in percent reaching clinically inactive disease is larger than zero).  With more imbalance, there is a 96% probability of showing that the biologic CTPs are more.

  Using the approach in Jung[27], we can calculate the approximate sample sizes needed for these analyses under a range of assumptions about the effect of biologic CTPs, the probability of CID in the non-biologic group and the degree of imbalance. Table 1 shows sample sizes giving 80% power with a one-sided α of 0.05.
