# Supplementary material for: Pilot study comparing the Childhood Arthritis & Rheumatology Research Alliance (CARRA) systemic Juvenile Idiopathic Arthritis Consensus Treatment Plans
Source: Pediatr Rheumatol Online J. 2017 Apr 11;15:23. doi: 10.1186/s12969-017-0157-1 (PMC5387287; doi:10.1186/s12969-017-0157-1)
Supplement: Supplementary file 2 — Scenarios evaluated for degree of imbalance in propensity score modeling. (DOC 28 kb) [file 12969_2017_157_MOESM2_ESM.doc]

**Additional file 2**

**Table S1**: Scenarios evaluated for degree of imbalance in propensity score modeling.

|  |  |  |  |  |  | P(CID) GC= 0.30 | | | P(CID) GC= 0.20 | | | |  | |
| --- | --- | --- | --- | --- | --- | --- | --- | --- | --- | --- | --- | --- | --- | --- |
|  | Propensity Score Quintile | | | | |  | P(CID) in Biologic CTP | | | |  |  | |  |
| Imbalance | Q1 | Q2 | Q3 | Q4 | Q5 | 0.45 | 0.50 | 0.55 | 0.35 | 0.40 | 0.45 |  | |  |
| Small | 20:80 | 27:73 | 33:67 | 40:60 | 46/54 | 311 | 177 | 114 | 268 | 157 | 104 |  | |  |
| Medium | 13:87 | 23:77 | 33:67 | 43:57 | 53/47 | 330 | 188 | 121 | 287 | 168 | 111 |  | |  |
| Large | 3:97 | 18:82 | 33:67 | 48:52 | 63/37 | 380 | 216 | 138 | 334 | 194 | 128 |  | |  |
